# Functional and Structural Brain Connectivity in Children With Bilateral Cerebral Palsy Compared to Age-Related Controls and in Response to Intensive Rapid-Reciprocal Leg Training

**DOI:** 10.3389/fresc.2022.811509

**Published:** 2022-04-05

**Authors:** Diane L. Damiano, James J. Pekar, Susumu Mori, Andreia Vasconcellos Faria, X. Ye, Elaine Stashinko, Christopher J. Stanley, Katharine E. Alter, Alec H. Hoon, Eric M. Chin

**Affiliations:** ^1^Department of Rehabilitation Medicine, NIH, Bethesda, MD, United States; ^2^FM Kirby Research Center for Functional Brain Imaging, Kennedy Krieger Institute, Baltimore, MD, United States; ^3^Russell H. Morgan Department of Radiology and Radiological Science, Johns Hopkins School of Medicine, Baltimore, MD, United States; ^4^Johns Hopkins School of Medicine, Baltimore, MD, United States; ^5^Kennedy Krieger Institute, Baltimore, MD, United States

**Keywords:** imaging, spastic diplegia, plasticity, brain volumes, physical therapy, brain imaging

## Abstract

**Background:**

Compared to unilateral cerebral palsy (CP), less is known about brain reorganization and plasticity in bilateral CP especially in relation or response to motor training. The few trials that reported brain imaging results alongside functional outcomes include a handful of studies in unilateral CP, and one pilot trial of three children with bilateral CP. This study is the first locomotor training randomized controlled trial (RCT) in bilateral CP to our knowledge reporting brain imaging outcomes.

**Methods:**

Objective was to compare MRI brain volumes, resting state connectivity and white matter integrity using DTI in children with bilateral CP with PVL and preterm birth history (<34 weeks), to age-related controls, and from an RCT of intensive 12 week rapid-reciprocal locomotor training using an elliptical or motor-assisted cycle. We hypothesized that connectivity in CP compared to controls would be greater across sensorimotor-related brain regions and that functional (resting state) and structural (fractional anisotropy) connectivity would improve post intervention. We further anticipated that baseline and post-intervention imaging and functional measures would correlate.

**Results:**

Images were acquired with a 3T MRI scanner for 16/27 children with CP in the trial, and 18 controls. No conclusive evidence of training-induced neuroplastic effects were seen. However, analysis of shared variance revealed that greater increases in precentral gyrus connectivity with the thalamus and pons may be associated with larger improvements in the trained device speed. Exploratory analyses also revealed interesting potential relationships between brain integrity and multiple functional outcomes in CP, with functional connectivity between the motor cortex and midbrain showing the strongest potential relationship with mobility. Decreased posterior white matter, corpus callosum and thalamic volumes, and FA in the posterior thalamic radiation were the most prominent group differences with corticospinal tract differences notably not found.

**Conclusions:**

Results reinforce the involvement of sensory-related brain areas in bilateral CP. Given the wide individual variability in imaging results and clinical responses to training, a greater focus on neural and other mechanisms related to better or worse outcomes is recommended to enhance rehabilitation results on a patient vs. group level.

## Introduction

Cerebral palsy is a heterogeneous group of disorders that affect movement and posture, resulting from dysgenesis or injury in the developing brain (1). Brain magnetic resonance imaging (MRI) studies investigating structural integrity in children with cerebral palsy (CP) as compared to typically developing children have provided important insights into the specific pathways underlying disordered motor function in individuals with CP as well as insights into brain organization and plasticity during development or in response to interventions. Most studies have focused on unilateral (vs. bilateral) CP, the more functional subtype with less involuntary movements that affect scan quality. Even fewer studies have explored potential brain changes after motor training and their correlates with behavioral improvements.

Our primary aims here were to quantify changes in brain structure and connectivity in a homogeneous group of children with bilateral CP, all preterm with diffuse white matter injury, before and after intensive locomotor-based training, and the relationships of these with changes in mobility. As an initial step, baseline brain measures in our CP cohort were compared to those from an age-related cohort of children with typical development to identify the most prominent brain abnormalities in our sample for comparison to the literature and as a baseline for assessing the direction of change post-training.

Diffusion tensor imaging (DTI) is a powerful technique used with increasing frequency in children with CP because it precisely identifies abnormalities in macro- and microstructure (2). Hoon et al. reported DTI results on 28 children born preterm all but one with bilateral spastic CP (3). Unexpectedly, in this cohort, injury in the posterior thalamic radiation (PTR) was more extensive than that in the corticospinal tracts (CST), based on qualitative assessment. Only the PTR findings were significantly associated with motor and sensory outcomes, suggesting that sensory tract injury may be more important in function than previously assumed. Yoshida and co-authors in the first quantitative analysis of a more diverse group of 26 children with bilateral and 8 with unilateral CP, with only half born preterm, instead found involvement of both tracts in CP that correlated with mobility and sensory function (4). A 2012 review of 22 DTI studies in CP by Scheck and co-authors, eight of which included only participants with bilateral CP and 5 more with a mixed but mainly bilateral cohort, (5) concluded that the CST tract was the most well studied and most consistently related to functional measures. The sensorimotor pathways showed similar results, although supported by less evidence. DTI has also been utilized to evaluate changes post intervention in bilateral CP after a 2-year intervention when all were receiving physical and occupational therapy in conjunction with autologous cord blood infusions and sham infusions on a random schedule. In a subset with positive functional changes, greater functional improvements were associated with greater increases in connectivity especially at higher intervention doses (6, 7).

In the first fMRI study to demonstrate neural plasticity after intervention in bilateral CP, increased functional brain activation was seen during an active ankle dorsiflexion task in three of six children with bilateral CP after a pilot trial of body weight supported treadmill training (8). It is important to note that movement artifacts which reduced their already small sample size by half are particularly problematic in CP. Diagnostic MRI often is done under sedation which is justifiable in clinical care. Given sedation risks, structural imaging for research purposes is typically done in sleeping infants or in awake children who are able to remain still for long periods. Active task-based functional imaging requires that the child be awake but still and able to comply with instructions. Hence, the majority of DTI or fMRI studies in CP have been in those with unilateral brain injury. Children with bilateral CP who have greater neurological involvement are more likely to have increased startle or other involuntary movements that may be exacerbated when asked to perform a motor task, precluding acquisition of high-quality images. Consequently, less is known about brain reorganization and plasticity in bilateral CP.

Resting state fMRI may be far more feasible than active task fMRI in this population since movement is not required. Further, intrinsic connectivity networks identified by this type of imaging and analysis have been shown to closely represent those activated by task performance or disrupted by pathology. The first resting state MRI connectivity analysis reported in bilateral CP found greater than normal connectivity across sensorimotor cortices in CP. They postulated that damage to the subplate neurons during the third trimester causes a reduction in thalamocortical inputs, hence less competition, and as a result, intracortical connections flourish (9).

For this investigation, we collected structural and resting state MRI and DTI data before and after an intensive locomotor-based training intervention in a group of ambulatory children, Gross Motor Function Classification System (GMFCS) Levels I-III, with bilateral CP, all of whom were born preterm, and in a same-aged cohort without CP at a single time point. The CP cohort studied here was a subset of those enrolled in a clinical trial where all were randomized to one of two equally-dosed exercise programs. The training paradigm was a 12-week in-home rapid, reciprocal lower limb exercise program on either a pediatric elliptical device or a motor-assisted stationary cycle with the specific goal of improving lower limb speed and coordination during the trained task and during walking. Once the target training speed was achieved, pedal resistance was gradually added to potentially enhance motor outcomes and magnify sensory afferent input to the sensorimotor brain regions. Since there were no differences found between the two devices in the functional outcome measures, the two groups were combined here to be used as correlates with the brain imaging outcomes (10).

The two primary study aims here were: (1) to investigate whether there were significant changes in structural and functional MRI/DTI measures after intensive locomotor training in those with CP and (2) to correlate individual changes in connectivity post-training with functional outcomes. Prior to the inclusion of the brain imaging data for those with CP, all were evaluated to ensure that diffuse white matter injury (DWMI) was the primary pathology.

Our primary hypotheses were that differences in functional and structural connectivity in the sensorimotor brain regions would be detectable as a result of training based on previous studies (6–8) and the degree of change would correlate with change in functional outcomes. Further, with the exception of interhemispheric sensorimotor connectivity which was expected to decrease post training and therefore show an inverse correlation with functional improvements (9), we predicted that greater connectivity post-training would be directly related to positive functional change.

Secondary aims were: (1) to quantify and compare brain volumes and connectivity in children with bilateral CP and typically developing controls as a basis for evaluating the type and magnitude of the abnormalities in our specific CP cohort and the direction of changes post-intervention; and (2) to correlate baseline functional measures and imaging results in those with CP, for comparison with previous studies. We anticipated that brain imaging group differences would be identified in both the CST and the PTR and that the degree of abnormality in baseline measures in CP would correlate directly with functional limitations.

## Methods

### Participants

Participants were a subset of children with cerebral palsy (CP) from a larger randomized clinical trial (NCT01086670) who met screening criteria for MRI and had data collected at baseline and after the 12-week intervention. We also collected MRI and DTI data at one time only on a group of healthy controls within the same age range using the identical scanner and imaging protocol. Inclusion criteria for both groups were ages 5–17 years inclusive, no contraindications to MRI, and ability to follow simple commands. Additional criteria for those with CP were: (1) diagnosis of bilateral CP with periventricular leukomalacia (PVL) as documented in the medical record; (2) preterm birth of 34 weeks of gestation or less; (3) GMFCS Level I-III; (4) no lower limb surgery within the past year or muscle injections within the past 4 months; (5) judged by parent or therapist as being able to complete an intensive training program; and 6) not currently participating in locomotor training.

The protocol (#10-CC-0073) was approved by the National Institutes of Health Institutional Review Board and written informed consent and assent were obtained for all participants prior to participation.

### Intervention Protocol

After enrollment, all children with CP were randomly assigned to training on either a pediatric elliptical device (Cardiokids, Kidsfit, Huger, South Carolina) or a computer-assisted cycle (MOTOmed gracile, RECK, Germany). The primary training goal was to progressively increase the speed of reciprocal leg movement. Once the child reached 40 RPMs on the assigned device, mild pedal resistance was added and gradually progressed as long as this did not lower training cadence. All were instructed to exercise for 20 min per day, 5 days a week, for 12 weeks and compliance was monitored on each device and with a written log. The dose of the intervention was chosen by reviewing previous locomotor training studies in CP with none >20 h which was then chosen as the target dose here. We had hypothesized that both intervention groups would improve significantly compared to a 12-week non-intervention period in device cadence and gait speed. We further hypothesized that changes would be significantly greater in the elliptical group because all four limbs were involved in training and the postural demands of this upright task were far greater and more closely resembled those during gait (i.e., more task-specific) [see Damiano et al. (10) for more details]. The results of the trial partly supported the first hypothesis in that each group improved significantly in the trained device cadence, but this did not transfer to significant improvements in cadence on the other device or to improved gait speed. The elliptical group failed to show superiority to the cycle group in either of these outcomes; therefore, we combined these data from both groups to be used here as correlates for brain imaging results.

### Data Collection

All children in the trial underwent three comprehensive assessments first at baseline, immediately post the 12-week intervention, and after a 12-week no intervention period. Due to limited availability of devices, half of the children underwent training immediately after baseline, with a final assessment after a 12-week no intervention follow-up period. The other half waited 12 weeks before starting training, so the second baseline was used as the pre-intervention assessment. Only the immediate pre- and post-training assessments in CP are reported here. The primary motor outcome measures were the pre- to post-training change in the assigned device cadence at a comfortable and as fast as possible pace, and free and fast gait speed. Secondary behavioral measures included: isokinetic knee extensor strength at 30 and 90 degrees per second, the Selective Control Assessment of the Lower Extremities (SCALE), two computer-based parent-report questionnaires, the PEDI-CAT (version 2.5) and the Pediatric Outcomes Data Collection Instrument (PODCI; version 1.36) each of which assesses the child's typical performance on everyday motor-related tasks.

### Brain Imaging Protocol

Participation in the imaging part of the study was optional for children enrolled with CP. Of the 27 participants with CP in the clinical trial, 22 agreed to participate and completed the screening form successfully with no contraindications to MRI. Each participant was asked to do the brain scans at all three assessment points; however, only the pre-intervention baseline and post-intervention data are reported here. We also recruited a control group of 19 children without CP within the same age ranges to participate in the same MRI scanning protocol at one time point, each also successfully completing the MRI screen. All females with child-bearing potential had a urine pregnancy test with a negative result the same day as the MRI scan.

During the scans, children were provided with ear protection and an MRI-screened parent was allowed to sit next to the child in the scanner if desired. The child was able to communicate the entire time with the person conducting the scan and was instructed to remain awake with eyes open and be as still as possible during each scan.

All data were collected on a Philips 3.0 T (Philips HealthCare, the Netherlands) Achieva scanner at NIH. This scanner used gradients running at 80 mT/m with a slew rate of 110 mT/m/s, and at 40 mT/m with a slew rate of 220 mT/m/s. The scanner was equipped with a thirty-two-channel receiver system and high-sensitivity multi-element receive-only head coils, allowing for parallel imaging, including partially parallel imaging using sensitivity encoding (SENSE) for faster scanning.

The type and order of the scans were as follows: T1, T2, FLAIR, resting state BOLD-fMRI, MPRAGE, and DTI. MPRAGE, resting state BOLD-fMRI, and DTI sequence data was used in this analysis. MPRAGE was performed with 1 mm isotropic resolution spanning the whole brain (matrix size 256 x 256 x 200 slices). This protocol was based on those used in routine clinical studies. BOLD-fMRI acquisition was performed in an awake resting state; i.e., participants were asked to try to remain awake with their eyes open and as still as possible for a period of 7 min. Functional data were collected using single-shot SENSE gradient-echo echo-planar imaging, sensitive to BOLD contrast, with TE = 30 ms, a flip angle = 70°, TR = 2.0 s, and 2.625 x 2.625 x 4 mm resolution (matrix size 96 x 96 x 37 slices covering the whole brain). A SENSE acceleration factor of 2.5 was used.

A single-shot EPI with SENSE acquisition was used for DTI. Echo time was 66 ms, and repetition time was >3 s. A SENSE reduction factor of 3 was used, which reduced the number of echoes in an echo train to 26. Diffusion-weighting was applied along 32 independent axes with b = 700 s/mm^2^ in addition to one b = 0 image. The imaging matrix was 256 x 256 with a field-of-view (FOV) of 211 x 211 mm, which gave 0.83 mm in-plane resolution. The slice orientation was axial with 2.2 mm thickness. Seventy slices covered the entire hemisphere and the brainstem. The scanning time for one complete DTI dataset was ~4.5 min. Total amount of time the child remained in the scanner was approximately 60 min.

### Image Processing and Analysis

#### Approach and Rationale

Raw anonymized data were analyzed at Kennedy Krieger Institute/Johns Hopkins University. Our attempts to identify neuroimaging-based signatures of response to intervention (Primary Aims 1 and 2) were designed considering two technical limitations:

1) Even within this etiologically- and functionally-selected cohort of children with CP, inter-individual neuroanatomical variation is substantial when compared to clinical populations in which quantitative analysis techniques have typically been used. Accurate registration (even on the resolution of regions of interest [ROIs]) has, until recently, been technically challenging (11).

2) Multimodal neuroimaging data are intrinsically high-dimensional, yet functional brain networks underlying gross motor function in neurotypical and clinical populations have not been fully specified (12). This limits the statistical power available to identify meaningful effects. particularly in small studies.

Acknowledging these challenges, we utilized a semi-automated ROI-level processing pipeline. Automated multi-atlas techniques have proven suitable for gray matter and white matter parcellation in clinical populations with anatomical variability (11); this (with manual validation) represented our first processing step. We focused attention upon brain regions clearly impacted by CP pathology (i.e., those with significant group-level differences in volume or in tissue microstructure when compared to typical controls per Secondary Aim 1) and upon regions with clear roles in motor functioning (primary motor cortex gray matter and corticospinal tract white matter). For context, we also examined correlations between baseline structural and functional imaging characteristics of these regions with functional status in children with CP (Secondary Aim 2). We examined changes in structural and functional imaging characteristics of these regions occurring in response to intervention (Primary Aim 1). We further examined correspondence between pre- to post-intervention changes in resting state functional connectivity associated with functional response to intervention (Primary Aim 2).

This prescriptive approach makes hypothesis testing feasible but does not permit identification of unexpected “off-target” brain network changes. To help inform future investigations, we extended functional connectivity analyses performed in Supplementary Aim 1 (comparison of children with CP vs. control children) and in Primary Aim 1 (changes pre- vs. post-intervention in children with CP) to examine functional connectivity throughout the brain. While clearly not powered for significance, we considered this exploratory analysis descriptive and reported results in terms of standardized effect sizes.

#### Assessment for Inclusion

Since the study was designed specifically to evaluate children with bilateral CP with diffuse periventricular white matter injury (DWMI), images were first reviewed to verify this. Two neurodevelopmental physicians (authors AHH and EMC) with CP and MRI analysis experience independently reviewed baseline MPRAGE and axial T_2_-weighted images using the MRI Classification System [MRICS; (13)]. Participants with CP were excluded if their primary imaging abnormality was not consistent with periventricular leukomalacia (category “B1”). Any disagreements between raters were resolved by discussion.

#### MPRAGE and DTI Processing

MPRAGE and DTI processing and segmentation were performed using the MRICloud automated processing platform (14). Briefly, MPRAGE segmentation in MRIcloud employs a multi-atlas labeling approach. Pre-processing includes orientation and homogeneity correction and two-level brain segmentation (a skull-stripping step followed by whole brain segmentation). Image mapping is then refined using a series of linear and nonlinear algorithms including large deformation diffeomorphic mapping (LDDMM). A final step applies label fusion based on a multi-atlas library (15). MRICloud computes volumes of each of 283 atlas regions, organized within a five-tiered hierarchical ontology (16) permitting analysis at multiple scales (e.g., at the level of cerebral hemispheres at the largest scale [level 1] and at the level of individual gyri at the smallest scale [level 5]).

Similarly, MRICloud performs DTI segmentation using diffeomorphic registration and multi-atlas fusion. Pre-processing includes tensor reconstruction and automated rejection of artifactual outliers (17). Diffeomorphic likelihood fusion to a multi-atlas library optimizes labeling based on mean diffusivity, fractional anisotropy (FA), and fiber angle maps (18). MRICloud computes scalar diffusion indices for white matter regions of interest. Of these, we use regional mean FA in this analysis.

Both MPRAGE and DTI segmentation results were individually assessed. Visually-apparent misregistration occurred only in scans with clear artifact (either motion- or susceptibility-related), and such scans were excluded from further analysis.

#### Resting State FMRI Processing

Resting state BOLD acquisitions were aligned to corresponding MPRAGE images and processed using AFNI 20.1.06. Specifically, we used AFNI-recommended pre-processing steps (including despiking, bandpass filtering from 0.01–0.1 Hz, blurring with a 4 mm Gaussian filter, and regression of nuisance components associated with motion parameters, with three principal signal components observed within a CSF mask, and from an additional component derived from signal within an eroded white matter mask) integrated with alignment to MPRAGE– permitting use of MRIcloud-derived ROIs for functional connectivity analysis. As per convention, the ROI-to-ROI functional connectivity is defined as the Fisher-transformed Pearson correlation between ROIs' BOLD time courses (19).

We used a more lenient motion censoring threshold than is typically used in adults without brain pathology. In children with CP who completed resting state BOLD scanning, 13 of 16 exhibited head motion in excess of the recommended AFNI threshold (>5 min scan time remaining after censoring of image frames with inter-frame displacement (IFD) in excess of 0.2 mm). As recommended by Power et al. (20), to identify a more appropriate threshold for this study, we empirically assessed distance-dependent deviations in inter-regional correlations, a hallmark of artifactual alterations in functional connectivity associated with head motion (21). Consistent with a previous report (22), we found (see Supplementary Material) that with use of other preprocessing steps reviewed above, no apparent distance-dependent bias was found at any threshold examined (IFD <0.2 mm, IFD < 0.3 mm, and IFD < 0.5 mm). As such we selected IFD < 0.5 mm as a compromise, thus permitting inclusion of most study participants while limiting bias introduced by head motion.

#### Imaging Features of Interest

As our study population consisted of individuals with bilateral motor deficits and bilateral brain abnormalities, we reduced data dimensionality in Primary Aims by combining pairs of lateralized ROIs in volumetric and DTI analyses. As discussed above, we further focused attention upon brain regions clearly impacted by CP pathology (i.e., those with significant group-level differences in volume or in tissue microstructure when compared to typical controls per Secondary Aim 1) and upon regions with clear roles in motor functioning (primary motor cortex gray matter and corticospinal tract white matter). For DTI analyses, we additionally conservatively limited analysis to large white matter ROIs consistently spanning volumes of at least 500 voxels. While not meeting this criterion, we also analyzed FA for corticospinal tracts due to their established role in motor functioning.

Combination of lateralized imaging features is not trivial when examining functional connectivity (defined by ROI-ROI pairs rather than by single ROIs). For hypothesis testing (examined between primary motor cortex and gray matter ROIs above), we averaged symmetric intra-hemispheric connection strengths. In particular, we defined


$$\begin{array}{l} {\text{ z}}_{\text{RegionA},\text{ RegionB}}^{\text{intrahemispheric}}\text{=}\\ \text{mean}({\text{z}}_{{\text{RegionA}}_{\text{Left}},{\text{RegionB}}_{\text{Left}}},{\text{z}}_{{\text{RegionA}}_{\text{Right}},\;{\text{RegionB}}_{\text{Right}}}) \end{array}$$

(where *z* indicates Fisher-transformed functional connectivity between two ROIs).

For exploratory analyses, we additionally examined homologous inter-hemispheric region pairs:


$$\begin{array}{l} {\text{z}}_{\text{RegionA}}^{\text{interhemispheric}}={\text{z}}_{\text{Region}{\text{A}}_{\text{Left}},\text{ Region}{\text{A}}_{\text{Right}}} \end{array}$$

For exploratory analysis including 55 lateralized ROIs, this results in examination of 1,485 intrahemispheric ROI-ROI pairs + 55 interhemispheric pairs.

### Statistical Analyses

#### Distributions and Normality

Multivariate normality testing (23) rejected normality of regional volumes both for children with CP as well as for controls (both *p* < 0.001). While Henze-Zirkler testing failed to reject normality of regional FA distributions (*p* = 0.24 and *p* = 0.21, respectively), non-parametric statistical tests were applied to both volumetric and FA data throughout for consistency. In contrast, Fisher transformation of region x regional functional connectivity metrics transforms functional connectivity into a near-normal distribution, permitting use of parametric statistics (24).

#### Behavioral Data

Even though the behavioral outcomes from the clinical trial have already been reported, we compiled these on the subset of those with CP included in the brain imaging results to illustrate their baseline characteristics (see Table 1) as well as to statistically compare the pre- and post-intervention results for this group. Paired *t*-tests were used to assess the effects of time (pre- vs. post-intervention) with *p* < 0.05 indicating significance (SPSS Version 23, IBM Corporation). Armonk NY). We also presented the primary outcome as a change in voluntary cadence on the device that was trained at each of the two speeds so that we could indicate the degree to which each participant changed as a result of the training in a single measure, rather than only evaluating the two devices separately as was done in the larger trial. These and all other change scores for the functional outcomes were computed for this cohort and correlated with the brain imaging outcome measures. It is important to note that the sample size for the clinical trial was powered on the primary motor outcomes and not the brain imaging outcomes.

**Table 1:** Participant demographic and baseline functional characteristics for the control group and CP imaging subgroups.

	**Controls**	**CP baseline**	**CP baseline**	**CP baseline**	**CP PRE-POST**	**CP PRE-POST**	**CP PRE-POST**
		**volumetric analysis**	**DTI analysis**	**rs-fMRI analysis**	**volumetric analysis**	**DTI analysis**	**rs-fMRI analysis**
# Participants	18	16	15	13	14	13	9
Age (mean ± SD)	13.3 ± 3.3	11.3 ± 3.4	11.3 ± 3.6	12.0 ± 3.2	11.6 ± 3.5	11.3 ± 3.5	12.3 ± 3.8
# female (%)	10 (56%)	13 (81%)	12 (80%)	11 (85%)	11 (79%)	10 (77%)	7 (78%)
GMFCS level (I,II,III)		(1,8,7)	(0,8,7)	(1,6,6)	(0,7,7)	(0,7,6)	(0,5,4)
# in elliptical group (%)		8 (50%)	8 (53%)	7 (54%)	8 (57%)	8 (62%)	5 (56%)
Elliptical (RPM free)		43 ± 34	49 ± 42	47 ± 33	48 ± 33	48 ± 32	56 ± 28
Cycle (RPM free)		96 ± 35	101 ± 31	99 ± 35	104 ± 35	99 ± 36	110 ± 30
Elliptical (RPM fast)		59 ± 48	63 ± 47	66 ± 47	67 ± 45	67 ± 47	81 ± 41
Cycle (RPM fast)		136 ± 60	141 ± 58	142 ± 60	144 ± 59	149 ± 58	160 ± 51
Gait velocity (free; m/s)		0.85 ± 0.28	0.86 ± 0.29	0.86 ± 0.29	0.87 ± 0.31	,83 ± 0.31	10.90 ± 0.28
Gait velocity (fast)		1.23 ± 0.25	1.26 ± 0.30	1.25 ± 0.28	1.29 ± 0.29	1.20 ± 0.29	1.21 ± 0.33
Gait cadence (free)		114 ± 24	114 ± 25	115 ± 25	113 ± 26	110 ± 23	117 ± 33
Gait cadence (fast)		145 ± 17	144 ± 17	146 ± 18	145 ± 17	139 ± 14	142 ± 16
KE Torque 30		0.74 ± 0.40	0.79 ± 0.41	0.77 ± 0.41	0.82 ± 0.40	0.78 ± 0.42	0.85 ± 0.49
KE Torque 90		0.46 ± 0.21	0.48 ± 0.22	0.47 ± 0.21	0.51 ± 0.20	0.48 ± 0.23	0.49 ± 0.27
SCALE score		9 ± 3	9 ± 3	9 ± 3	9 ± 3	9 ± 3	10 ± 4
PODCI Global Score		70 ± 14	72 ± 13	71 ± 13	72 ± 13	70 ± 14	75 ± 10
PODCI Transfers		77 ± 23	80 ± 21	80 ± 20	80 ± 26	76 ± 23	86 ± 11
PODCI Sports		48 ± 21	50 ± 29	49 ± 20	51 ± 19	48 ± 21	51 ± 20
PEDI Self-care		54 ± 9	57 ± 9	54 ± 9	55 ± 9	55 ± 9	57 ± 8
PEDI Mobility		48 ± 9	49 ± 9	49 ± 9	49 ± 10	48 ± 9	50 ± 6

*This table summarizes group-level characteristics (mean ± standard deviation for continuous variables; counts and percentages for categorical variables). Participants with CP tolerated MRI to varying degrees, and overlapping subsets of individuals were included in analyses (total included n = 16)*.

*GMFCS, Gross Motor Function Classification System; RPM, rotations per minute; m/s, meters per second; KE Torque, knee extension torque at 30 and at 90°/second; SCALE, Selective Control Assessment of the Lower Extremity (total score); PODCI, Pediatric Outcomes Data Collection Instrument scale standardized scores; PEDI, Pediatric Evaluation of Disability Inventory domain scaled scores*.

#### Brain Imaging Analyses and Relationships With Functional Measures

##### Primary Aim 1: Investigating Differences, If Any, in Imaging Features After Intensive Locomotor Training in Those With CP

For all individuals with CP who completed both pre- and post-intervention imaging, imaging features of interest (i.e. those significantly differing from control group characteristics in Secondary Aim 1 as well as features of regions preselected above) were compared using paired tests against a hypothesis of no change (signed-rank tests for regional volumes and FAs; *t*-tests for functional connectivity strengths). Bonferroni-Holm multiple comparisons correction (25) was applied (1, 4, 6, 10, and 18 volumetric comparisons at ontology levels 1, 2, 3, 4, and 5, respectively; 4 FA comparisons; and 3 functional connectivity comparisons).

##### Primary Aim 2: Correlate Individual Changes in Connectivity Post-training With Functional Outcomes

For individuals with both pre- and post-intervention functional imaging data, changes in functional connectivity were correlated with change scores in order to identify neural correlates of response to intervention. Spearman's ρ was used to assess correlation to avoid assuming linear relationships between variables. Correlations were assessed against a hypothesis of no correlation (ρ = 0; again with Bonferroni-Holm multiple comparisons correction applied). For results of interest, 95% confidence intervals for ρ values were computed using a bootstrap method over 1,000 iterations (the MATLAB bootci function). Note that MATLAB computes *p*-values of ρ values instead using exact permutation distributions, and so confidence intervals identified may be wider than one would expect based upon *p*-values (i.e., confidence intervals may include 0 despite a significant pre-correction *p*-value).

##### Secondary Aim 1: Comparing Regional Volumes, White Matter Microstructure, and Functional Connectivity Between Groups (Children With CP vs. Controls)

Raw (lateralized) pre-intervention imaging features for children with CP were compared groupwise with those of controls. Unpaired non-parametric rank sum tests were used to compare regional volumes and white matter regional FAs against a null hypothesis of equal groupwise medians. Bonferroni-Holm multiple comparisons correction (25) was applied for each category of imaging features (5, 18, 52, 70, and 198 volumetric comparisons at ontology levels 1, 2, 3, 4, and 5, respectively; 18 FA comparisons). The three functional connectivity assessments of interest were also compared (unpaired *t*-tests; Bonferroni-Holm correction for three comparisons).

##### Secondary Aim 2: Correlating Baseline Functional Measures in Those With CP With Baseline Imaging Features

In individuals with CP, imaging features of interest were correlated with pre-selected baseline functional measures (gross motor status (GMFCS), selective motor control (SCALE combined score), and baseline device cadences (RPM), gait velocity and cadence at freely-selected and fast speeds, isokinetic strength values, and scores from the PEDI-CAT and PODCI questionnaires, as well as against a covariate (age). Spearman's ρ was again used. Correlations were assessed against a hypothesis of no correlation (ρ = 0; again with Bonferroni-Holm multiple comparisons correction applied). For results of interest, 95% confidence intervals for ρ values were computed using a bootstrap method over 1,000 iterations (the MATLAB bootci function).

##### Exploratory Analyses

Functional connectivity analyses performed in Secondary Aim 1 (comparison of children with CP vs. control children) and in Primary Aim 1 (changes pre- vs. post-intervention in children with CP) were extended to bivariate gray matter ROI-ROI pairs throughout the brain. For each ROI-ROI pair, standardized effect size for each ROI-ROI pair was computed using unpaired Glass's Δ (a form of Cohen's d in which equal variance is not assumed).

## Results

### Baseline Group Characteristics

#### Non-imaging Characteristics

Twenty-two children (of 27 enrolled in the RCT) with CP completed at least one pre-intervention MRI scan. Of those, 18 of 22 demonstrated primary structural pathology consistent with periventricular leukomalacia (classification “B1” on the MRICS (13)). Three were excluded for having primary structural pathology consistent with a brain malformation (MRICS classification “A”), and one had a large circumscribed area of parenchymal loss consistent with resection (MRICS classification “D”). Two individuals with CP and one control were also excluded from analysis: One with CP had persistent motion artifacts impacting all MRI sequences, the other two had susceptibility artifacts from metallic dental braces.

Demographic and performance characteristics of the remaining 16 individuals with CP and 18 typically-developing control participants are summarized in Table 1. Age and sex distributions did not differ significantly between the study group and the control group [mean age 11.3 for CP and 13.3 for Control (*p* = 0.09 by *t*-test); 81 vs. 56% female (*p* = 0.22 by chi-squared test)]. Mean ages of those with CP for each of the smaller imaging subgroups were the same or higher (closer to Control mean age) than for the larger CP group of 16.

#### Group-Level Behavioral Characteristics

The paired t-tests comparing pre- and post- functional outcomes were run on the group of 13 participants with CP who had pre-post DTI data; and then again on the group of 9 participants with pre-post resting state MRI data. In both cases, only the trained device cadence had increased significantly as a result of the intervention; the change at the self-selected pace was 27 and 31 RPMs (*p* < 0.01) and at the fast speed was 45 and 41 RPMs (*p* < 0.01), for the DTI and MRI subsets, respectively. In the DTI subset, the change in freely selected velocity of 1.0 m/s showed a non-significant trend (*p* = 0.052).

#### Brain Imaging Analyses

Even though the comparison of CP and controls is a Secondary Aim (#1), these will be discussed first since the pre-post intervention results that address the two Primary Aims in the group with CP are presented with respect to the observed differences between cohorts.

#### Volumetric Imaging Characteristics

**Figure 1:**
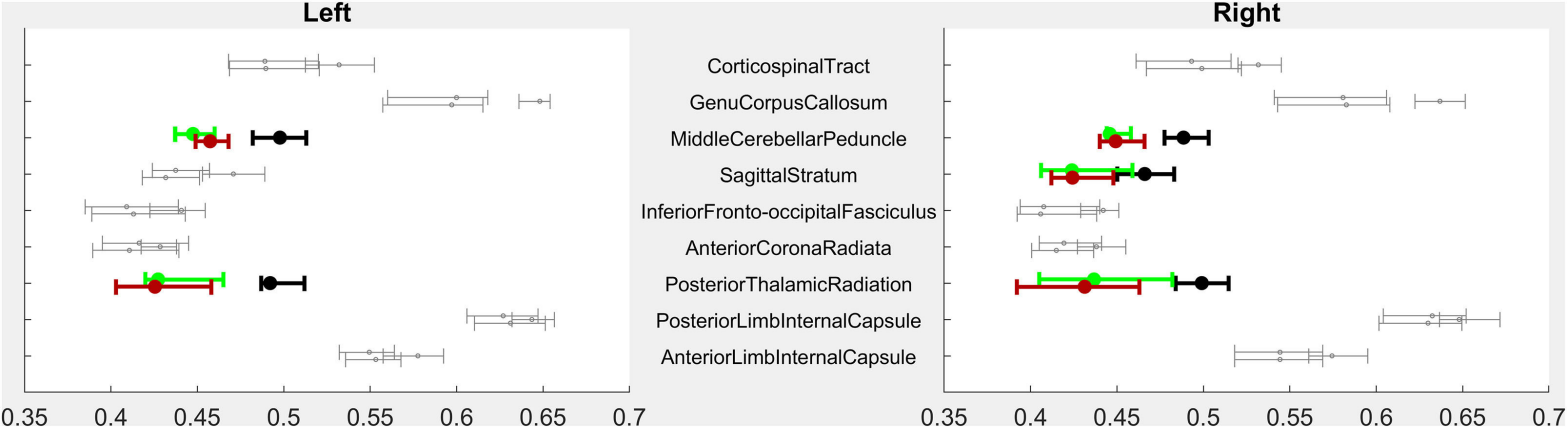
PRE- vs. POST-intervention group-level imaging characteristics–Volumetric analysis. For each region of interest (ROI), error bars indicate median and 95% confidence intervals of ROI volumes for children with CP PRE-intervention (highest of each triplet), POST-intervention (lowest of each triplet), and CTRL children (middle of each triplet). X-axis volumes are normalized in terms of % difference from median CTRL ROI volume. Bolded, colored triplets of error bars (CP-PRE = green; CTRL = black; CP-POST = red) indicate ROIs with significant CP-PRE vs. CTRL group differences surviving Bonferroni-Holm correction (53 ROIs compared in Level 3-resolution analysis). See Supplementary Figure for full Level 1–5 analyses. ROI designations reflect those of the MRIcloud MPRAGE atlas (19).

As a group, children with CP had significantly smaller parenchymal volumes than typically-developing children in subcortical white matter structures but also in deep gray structures including the thalami, the midbrain, and the pons (Figure 1). Children with CP had lower volumes within white matter posteriorly (40% less volume than in Control children bilaterally), anteriorly on the right (21% lower), in limbic white matter on the left (16% lower), and in the corpus callosum (25% lower). Volume was lower in left anterior white matter (19% lower) and right limbic white matter (14%) as well, but these comparisons did not reach significance. Decreased volume was also seen in the thalamus (30% lower), the midbrain (19% lower), and the pons (24% lower).

#### White Matter Microstructural Imaging Characteristics

**Figure 2:**
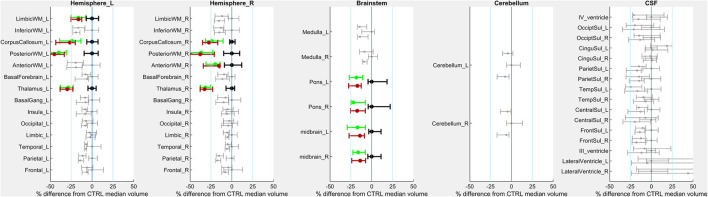
PRE- vs. POST-intervention group-level imaging characteristics–Fractional anisotropy. For each region of interest (ROI), error bars indicate mean and 95% confidence intervals of ROI volumes for children with CP PRE-intervention (highest of each triplet), POST-intervention (lowest of each triplet) and CTRL children (middle of each triplet). The X-axis indicates regional FA. Bolded, colored triplets of error bars (CP-PRE = green; CTRL = black; CP-POST = red) indicate ROIs with significant CP-PRE vs. CTRL group differences surviving Bonferroni-Holm correction (18 ROIs compared).

Fifteen children with CP and all 18 Controls completed baseline diffusion tensor imaging (DTI). As a group, children with CP had significantly lower mean FA within bilateral posterior thalamic radiations, sagittal striata, and middle cerebellar peduncles (Figure 2).

#### Resting-State BOLD Functional Connectivity

All children who underwent baseline MRI completed the 7-min resting-state BOLD sequence. However, three individuals with CP (*n* = 13 included) and one Control (*n* = 17 included) had inter-frame motion in excess of 0.5 mm for >2 min of scan duration precluding analysis.

We primarily examined functional connectivity of primary motor cortex (the precentral gyrus) and primary sensory cortex (postcentral gyrus) to gray matter ROIs impacted in structural analyses, i.e., the thalamus, the midbrain, and the pons. None of these three connections showed a significant groupwise difference.

**Figure 3:**
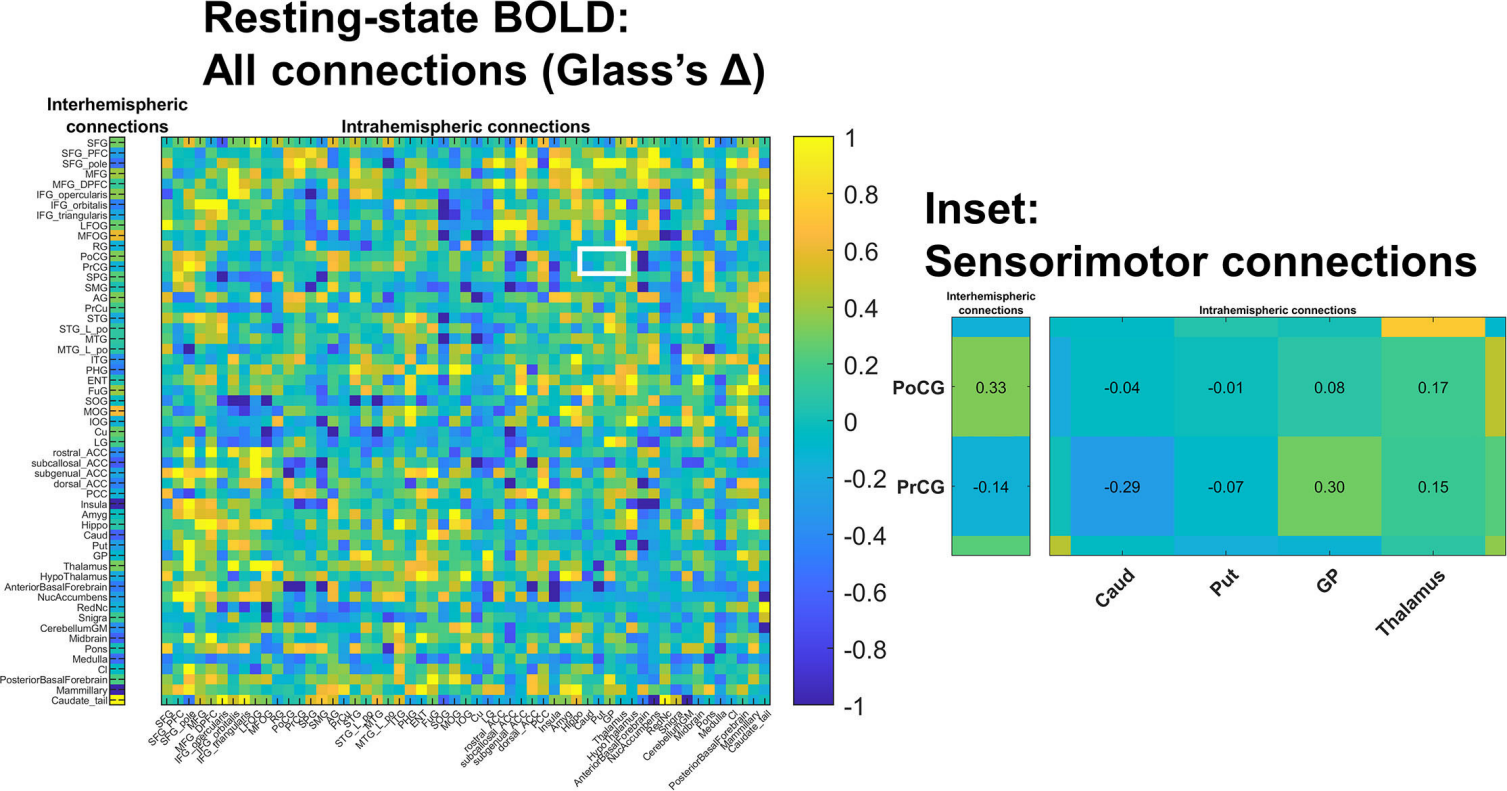
Changes in resting state BOLD functional connectivity: All connections. Functional connectivity was assessed in a non-selective manner including both *intra*-hemispheric connections **(right)** as well as *inter*-hemispheric connections **(left)**. No differences survived multiple comparisons correction; colors indicate estimated standard effect size (Glass's Δ). The white-outlined rectangle highlights cortical-deep gray sensorimotor connections (inset); estimated Δ values are shown in inset cells. Groupwise functional connectivity in sensorimotor connections did not grossly change PRE- vs. POST-intervention.

We also examined the interhemispheric connectivity between the precentral and postcentral gyri to specifically address whether connectivity was greater than normal as hypothesized. No significant differences were found, with the mean values in the post-central gyrus nearly identical across groups. However, the Glass Δ effect size for the precentral gyrus connectivity in CP vs. Controls was found to be moderately high and positive (Δ = 0.61; CI [−0.13 to +1.38]) (Figure 3).

### Group-Level PRE- and POST-intervention Imaging Characteristics

Fourteen of 16 children with CP who completed PRE-intervention MRI also completed the 3-D MPRAGE MRI sequence amenable to volumetric analysis POST-intervention.

There was a trend toward increased volume in the midbrain (+111 mm^3^ [95% CI: +18 to +146mm^3^]; corrected *p* = 0.054). Increases were small and represented ~1% of median volume seen in the control group.

Thirteen children with CP (all but one child analyzed above) completed DTI both PRE- and POST-intervention. There were no detectable differences in mean FA as a result of the intervention.

Nine children with CP completed resting state fMRI with acceptable levels of head motion both PRE- and POST-intervention. There were no detectable differences in functional connectivity.

#### Correlations Between Changes in Functional Connectivity and Response to Intervention

For the nine children who completed both PRE- and POST-intervention BOLD imaging, we found no correlation between change in functional connectivity measures (POST- minus PRE-intervention) and the primary outcome measure (change in trained-device cadence).

**Figure 4:**
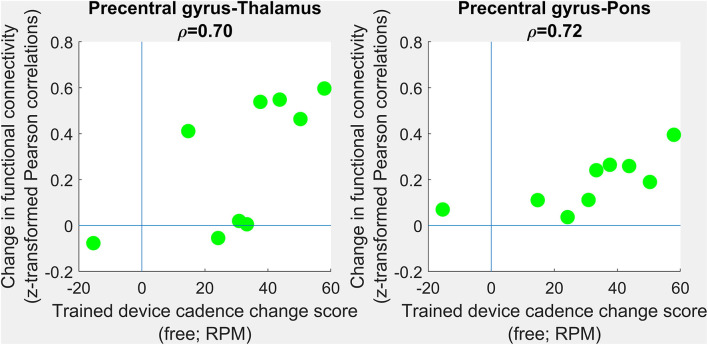
Correlations between changes in functional connectivity and response to intervention. PRE- to POST-intervention changes in functional connectivity were assessed in terms of association with PRE- to POST-intervention improvements in trained-device cadence. No group-level differences survived multiple comparisons correction. Scatterplots show trends toward improvements in free cadence associated with increases in precentral gray matter-to-thalamus **(left)** and precentral gray matter-to-pons **(right)** functional connectivity. Each green circle corresponding to one individual.

No correlations survived multiple comparisons correction. However, large positive correlations (Figure 4) were observed between changes in trained modality cadence (free speed) and changes in precentral gyrus-thalamus functional connectivity (ρ =0.70; 95% CI 0.44–1.00; corrected *p* = 0.12) and changes in precentral gyrus-pons functional connectivity (ρ =0.72; 95% CI −0.28–1.00; corrected *p* = 0.08). Interhemispheric connectivity in the precentral and postcentral gyri showed negligible change.

We conducted a more exploratory examination of interregional functional connectivity within inter- and intrahemispheric ROI pairs in terms of standardized effect size (Glass's Δ). Absolute values of at least 0.8 are considered “large” effect sizes in behavioral science, and absolute values of at least 0.5 are considered “medium” effect sizes (26). Connectivity between primary sensorimotor cortex (pre- and postcentral gyrus) and deep gray nuclei (caudate, putamen, globus pallidus, and to a lesser degree thalamus) trended higher in children with CP (Supplementary Figure). Effect sizes were large between: postcentral gyrus and caudate (Δ=1.17; 95% confidence interval 0.34–2.06); postcentral gyrus and putamen [Δ=1.02 (0.22-1.87)]; postcentral gyrus and globus pallidus [Δ=0.83 (0.06–1.64)]; precentral gyrus and putamen [Δ=0.80 (0.04–1.61)].

#### Correlation With Functional Measures and Covariates

Thirty-nine symmetric ROIs with significant between-group volume differences (one ROI, brainstem, at the most coarse scale of parcellation, and 4, 6, 8, and 20 ROIs at progressively more refined scales) were further examined for correlation with functional measures. The three symmetric white matter ROIs with significant group differences in FA (posterior thalamic radiations, sagittal striata, and the middle cerebellar peduncles) were also examined. See the Supplementary Tables for full reporting of correlation strengths.

In volumetric and DTI analyses, only associations with age remained following multiple comparisons correction. Older age was associated with larger volume in anterior white matter regions (ρ = +0.72), specifically in the superior corona radiata (ρ = +0.79) within anterior deep periventricular white matter (ρ = +0.72). Older age was also associated with greater volume in the posterior limb of the internal capsule (ρ = +0.77). Similarly, mean FA was positively correlated with age in the sagittal striatum (ρ = +0.63) and in the corticospinal tract (ρ = +0.74).

No volumetric or DTI features correlated significantly with PRE-intervention functional measures following multiple comparisons correction. *Post-hoc*, we examined bulk trends associated with preserved regional white and gray matter volumes, respectively. Of (17 functional measures) x (11 Level 5 white matter ROIs) = 187 correlations examined, 147 (79%) were positive (Wilcoxon sign test *p* < 10^−14^ against a hypothesis of no trend). Of (17 functional measures) x (7 Level 5 gray matter ROIs) = 119 correlations examined, 59 (50%) were positive (*p* > 0.5).

In resting-state functional connectivity analysis, higher PRE-intervention precentral gyrus-midbrain connectivity was associated with better mobility (ρ = +0.69 (0.10 to 0.91) vs. PEDI Mobility T-scores; corrected *p* = 0.028). Additional associations did not reach significance following correction.

## Discussion

The primary purpose of the clinical trial which was the source of these data was to improve mobility in a fairly homogeneous group of children with bilateral CP, all born preterm and having DWMI. In high income countries, the etiology in approximately 30–50% of children with CP is related to injuries in the infant born preterm (27, 28), so this is clearly an important sub-group for study in CP. Specifically, we aimed to improve reciprocal coordination as evidenced by greater movement speed in the lower limbs during the trained locomotor task (cycle or elliptical) and potentially to improve gait speed and mobility in general. In the larger trial and in the subset here, device speed improved dramatically and significantly at both a freely-selected and fast pace. However, these did not translate to improvements in other motor tasks on the group level. Individual outcomes showed considerable variability, with some showing large improvements in gait speed, most showing a negligible effect and some even showing a slower speed after training, with no clear explanation emerging to explain the individual differences in outcomes.

The current study was focused on the brain imaging results from the pre- and post-intervention assessments for those from the RCT on whom we were able to obtain images of sufficient quality for structural MRI (for regional volumes), DTI (for FA), and rsMRI (connectivity). For this part of the study only, we additionally recruited age-matched controls to provide a basis for detecting abnormalities in our clinical cohort. The two primary aims were to determine whether there were measurable changes in brain imaging parameters as a result of training on a group level and whether there were associations between motor and brain outcomes within individual participants with CP. Secondary aims were to utilize baseline brain measures to characterize the cohort studied here compared to controls within the same age range and to evaluate whether baseline measures of brain structure or function correlated with baseline motor status.

### Primary Aims 1 and 2

Explore whether there were group level changes in brain imaging parameters as a result of training and whether individual changes in brain structure or function as a result of training were associated with changes in motor outcomes.

We did not anticipate that large scale structural changes in the brain would be evident after training which had a maximum total dose of 20 h, but alterations in connectivity patterns were hypothesized. However, in contrast to our expectations, the only non-significant trend were changes in midbrain volumes that may have been related to developmental changes in white matter tracts analogous to those seen over time in typically-developing children (29) rather than in response to training. No changes in structural or functional connectivity were found at the group level. Because we had hypothesized the increased interhemispheric connectivity in the sensorimotor regions would reduce with reciprocal interlimb training, we examined this specifically for trends. While the CP mean across precentral gyrus compared to Controls showed a moderate positive effect size that indicates some support for earlier findings, changes in this in response to training were negligible. The smaller sample and the lack of significant functional changes beyond the task-specific ones were limitations here. Although the intervention used devices that simulated locomotor functions that were presumed to enable transfer to walking, this was not shown to be the case. Perhaps speeded gait training would have produced more clinically relevant change in mobility; however, brain outcomes would not necessarily be expected to differ if similar intensities. Far more research needs to be done to address the critical question on the types of interventions and doses that can improve brain-behavior outcomes for children with CP and those with other brain injuries.

Two reports [Bleyenheuft et al. (30) and Araneda et al. (31)] both which evaluated brain outcomes after an intensive combined upper and lower limb training in those with unilateral CP did demonstrate positive changes in both hemispheres (changes in laterality toward greater contralateral control) that correlated with changes in upper limb function only, even though the lower limb was also trained. Araneda et al. noted that changes in both brain and function were quite inconsistent across participants even in a task-evoked paradigm as opposed to the resting state paradigm reported here. Given the variability in outcomes also seen in our sample, we performed a more exploratory evaluation of relationships by examining those with non-significant correlations but large associations (~50% shared variance). These very preliminary results seem to suggest that increases in pre-central gyrus connectivity with the thalamus and pre-central gyrus connectivity with the pons may be associated with improvements in the free speed primary outcome. The results from the unilateral CP study, similar to these very preliminary findings, seem to indicate that brain changes in CP in the direction of greater similarity to controls can occur in parallel with motor improvements, providing promising evidence of neuroplasticity as a result of motor training in CP. The training dose in both of these reports was 90 h, which was 4.5 times higher than the maximum dose used here of 20 h, and is a likely explanation for their more pronounced changes. Clearly, more studies are needed that explore the neural correlates of functional motor changes and the dose-response relationships.

### Secondary Aims 1 and 2

Evaluate differences in our CP cohort with periventricular white matter injury compared to an age-related cohort without CP and how these correlate with the level of motor disability in those with bilateral CP.

Due to the small sample size and narrow etiology (preterm with PVL) and limited severity range of our cohort (only GMFCS Levels I-III), differences between the CP and control groups may not be as pronounced as in some other studies; however, the major differences found here are largely similar to other reports. Lee et al. (32) in their cohort of 43 participants with bilateral spastic CP and PVL, all of whom were born preterm before 34 weeks of gestation (identical to our inclusion criteria), found decreased white matter volume in the PTR, CC, and the corona radiata and with respect to the gray matter, decreased volumes in the sensorimotor cortex, posterior cingulate cortex, the parietal temporal and occipital lobes, and in the basal ganglia, thalami and the cerebellum. The greatest difference from controls in our study was in white matter (WM) volume which is not surprising since this is the defining pathology in our cohort. However, Bax et al. (33) cautioned that white matter injury may not be detected in nearly 30% of those with bilateral CP on conventional MRI. The most pronounced difference in our cohort was in the posterior WM (40% less in CP) with the anterior differences only significant on the right. The corpus callosum volume which connects the WM tracts across hemispheres was also significantly less in CP. Thalamic volume was 30% less in CP with lesser differences also in the Midbrain and Pons. Valkama et al. (34) reported decreased brainstem volume in cross-section in those with PVL, pronounced especially the pons, because of the diminished fiber numbers in the central white matter tracts. (32) Microstructure analyses confirmed group differences for the posterior thalamic radiation (PTR) but differences were not found here for the corticospinal tract (CST).

It is important to note that this pattern of structural imaging sequelae (decreased volume in white matter tracts and in deep gray nuclei with less prominent differences in white matter microstructure) has also been seen in children born preterm without PVL (35) though to a substantially lesser degree (e.g., an average 7% decrease in subcortical gray matter volume in (36) as opposed to the ≥19% decreases seen in this study). Generally, when assessed in early development gray matter differences are most prominent, while later in childhood, white matter differences may become more apparent (37). With respect to general differences in brain connectivity between preterm infants and full-term controls, the thalamocortical circuits tend to be the most vulnerable, as shown by higher than normal diffusivity measures with FA results less clear or consistent (38). Thus, our findings here are at least partly attributable to preterm birth alone; although the more definitive FA results for the PTR here may more clearly differentiate our CP cohort from a healthy preterm one. In contrast to the thalamocortical circuits, effects on other regions emerge in association with brain injury on preterm infants, especially in the CST where lower FA is a prominent finding with more abnormal values associated with greater functional severity (38). The lack of significance for the mean CST FA differences here and in other studies remains incompletely understood; however, our small sample size was likely one factor here.

We explored a larger number and range of functional correlates compared to other studies, many of which had mainly utilized the GMFCS, and likely due in part to limited power, found no significant relationships with MRI or DTI measures and motor function when we corrected for multiple comparisons. Given this limitation, the lack of significant findings here is largely inconclusive.

In a more exploratory analysis in which we did not correct for multiple comparisons but instead examined variance explained, interesting but less conclusive patterns emerged. The most functionally relevant parameter was the resting state connectivity of the precentral gyrus with the midbrain which showed high levels of shared variance with all of the patient-reported measures of motor function, with the PEDI Mobility measure showing a strong and significant relationship. The midbrain plays a major role in motor control, impairment of which is the hallmark of CP, with the connection between the motor cortical regions and the spinal cord central to this.

Correlations between brain imaging parameters show a wide range of results across studies. Lee et al. (32) found in bilateral CP with PVL that white matter volume was not associated with motor function; however, decreased volume of the pre- and post-central gyri tended to be associated with greater motor severity as measured by GMFCS. In their microstructure analyses they sought to address the controversy over whether the damage to the CST tracts or thalamocortical circuits was more predominant and more highly related to the motor disorder. They contended that their voxel-based microstructure analyses were more sensitive than region of interest-based approaches and reported that the bilateral CST and the corpus callosum measures were most highly related to the functional severity as measured solely by GMFCS, though thalamo-cortical connectivity also significantly correlated with functional severity. They included all GMFCS levels in their study, which differed from this study, but was more similar to the sample distributions in Hoon et al. (3) and Yoshida et al. (4) While Lee and Yoshida reported differences in both the CST and the PTR with both correlated with function and stronger with the CST, using two different quantitative analyses, Hoon and our results failed to find CST differences or significant correlations of the CST with function. GMFCS correlations were not found in our study, but here range truncation (only GMFCS level I-III with only one participant in level I) was a likely explanation for this result. Cho et al. (39) also reported no GMFCS correlations with DTI in a much larger but more heterogeneous cohort of children with CP and PVL, again in contrast to results from other studies using quantitative DTI analyses.

Kersbergen et al. (40) evaluated changes over time in high risk preterm infants in the CST and thalamic volume. They noted that while abnormalities in the CST preceded those found in the thalamus, suggesting that disruptions to the thalamus were more downstream, they concluded that thalamic volume and not CST integrity was a significant predictor of CP severity. Arrigoni et al. (41) performed both ROI- and voxel-based whole brain DTI analyses on 25 children with bilateral CP and PVL. Noting that both methods produce similar results, they reported that the largest differences from controls were found in the PTR with z-scores of −3.6 and −3.0 for the left and right, respectively and the CC which also had z-scores ≥-3.0. The left and right corticospinal tracts also differed significantly but with lower z-scores of −1.5 and −2.1 respectively. Both were significantly negatively correlated with GMFCS levels (lower FA related to poorer mobility).

Jiang et al. (42) noted that CST damage had high specificity but lower sensitivity for diagnostic purposes. CST involvement can be seen in multiple CP subtypes to varying degrees and in some preterm infants who never develop CP, which can weaken its accuracy as a predictor. Of note, in the follow-up study by Jiang et al. (43), more global network-based evaluation of cortical structural connectivity improved prediction sensitivity and specificity. Individual differences in CST involvement as seen here and in other reports alone may lead to differences across studies, especially those with smaller and/or more variable samples. One important conclusion from these and other studies (5) is that CST damage should not necessarily be presumed as the sole cause of the motor deficits in an individual child with CP and that sensory tract involvement should also always be considered. A more nuanced understanding of the interactions between sensory and motor pathways and regions during early development in those with PVL and the implications of differing individual profiles on prognosis and optimal rehabilitation prescription remain important research goals in CP.

Investigators are beginning to look beyond MRI-based measures for addressing these and other questions to more mobile and accessible neuroimaging modalities such as EEG (electroencephalography) and fNIRS (functional near infrared spectroscopy). These technologies are able to assess functional brain activations in more naturalistic settings and artifacts. These have the potential to greatly expand our knowledge base on cortical dynamics during simple and complex motor tasks including gait in children with CP (44, 45) and to serve as outcome measures for clinical trials of individuals with CP or stroke (46). While more technically challenging, optimal use of research MRI in this population may lie in its potential to integrate structural and functional brain measures on an individual basis (co-registration) and its ability to assess deep brain structures not easily accessible using scalp surface-based neuroimaging modalities.

Larger datasets using sophisticated quantitative measures of brain structure will not completely resolve the discrepancies across studies reported here, e.g., on which brain regions seem to be most prominently affected and how these relate to function, because a major source of the variability lies within the individuals who comprise the study cohort. Large group analyses can inform us of the range of findings in a specific population or sub-population, and even their estimated prevalence, but will not be able to provide an individual prognosis or prescribe the optimal intervention plan. Weinstein et al. (47) made the astute observation that each child with CP has an individualized response based not only on the pattern and extent of their initial brain injury but also on a multitude of additional genetic and experiential factors. Wide variation in rehabilitation outcomes in CP even within similar subtypes is seen across intervention types, including highly recommended therapies such as intensive upper limb training. This challenges the field to more closely examine responders vs. non-responders to interventions, and ultimately achieve a more individualized (personalized) level of care (48). Variability in training outcomes in CP is strikingly similar to the individual variability observed in responses to medication, suggesting that individual genetic variations may be similarly important in neurorehabilitation. These may influence the differences in outcomes from preterm birth or hypoxic-ischemic or other perinatal insults as well as influencing a child's ability to learn new motor skills (49, 50). Developing individualized prescriptions from a child's brain imaging results are not yet possible, but should be an aspirational goal of future research.

Limitations of this study as noted previously were the small sample size and narrow GMFCS range; however, this was the first RCT evaluating a comprehensive set of brain and motor outcomes from locomotor training in CP, and only the second, and larger, study examining neuroplasticity in bilateral CP (8). Another limitation is that slightly different sample sizes were used across imaging modalities, with resting state fMRI being the most challenging data to obtain, and therefore results across modalities may not be as comparable as using the same cohort for each. However, our study, as with others in CP reporting on multiple brain imaging techniques [e.g., Lee et al. (32), Weinstein et al. (47)] chose to maximize the size of each dataset rather than restrict numbers on all to the lowest common subject group. Best practices for MRI acquisition have evolved since this study was conceived. Differences (e.g., use of anisotropic voxel size for DTI acquisition) may introduce biases when comparing absolute values to those acquired using modern standardized protocols [e.g., artifactual reduction of FA in ROIs containing fiber crossings (51)]. Longer resting state acquisitions are now recommended for more robust connectivity assessments (52). We did use a more expansive set of motor outcomes as correlates although this further limited power and failed to provide any significant new findings. However, extending outcomes beyond GMFCS, which was most commonly used here as a measure of motor involvement, is imperative when examining neuroplasticity after training, since the GMFCS is not an evaluative measure and is unlikely to change in response to training.

## Conclusions

This study supports previous reports on significant involvement of the sensory tracts in CP which is primarily, but not exclusively, a motor disorder. No conclusive evidence of neuroplastic effects as a result of training were seen. However, an effect size analysis of shared variance revealed that increases in pre-central gyrus connectivity with the thalamus and the pons may be associated with improvements in the trained device free speed primary outcome. Exploratory (uncorrected) analyses also revealed some interesting potential relationships between brain integrity and multiple functional outcomes in CP, with the functional connectivity between the motor cortex and midbrain showing the strongest potential relationship with mobility in everyday life. Increased focus on responder and non-responder sub-group or individual brain findings and their correlates is recommended to advance neurorehabilitation practice and outcomes.

## Data Availability Statement

The raw data supporting the conclusions of this article will be made available by the authors, without undue reservation.

## Ethics Statement

The studies involving human participants were reviewed and approved by the National Institutes of Health Institutional Review Board. Written informed consent to participate in this study was provided by the participants' legal guardian/next of kin.

## Author Contributions

DD: study conception, data analysis, and interpretation. JP, SM, and AF: development of imaging protocol and analytic techniques. ES: data interpretation. XY: imaging and statistical analyses and interpretation. CS: study coordination, data collection, management, and analysis. KA: participant recruitment, data collection, and interpretation. AH: development of imaging protocol and analytic techniques, data analysis, and interpretation. EC: performed imaging and statistical analysis, data analysis, presentation, and interpretation. All authors contributed to the article and approved the submitted version.

## Funding

This work was funded by the Intramural Research Program of the National Institutes of Health Clinical Center (Protocol # 10-CC-0073) and in part by the Cerebral Palsy Alliance Research Foundation (PG16617) and the Johns Hopkins Neurosurgery Pain Research Institute.

## Conflict of Interest

The authors declare that the research was conducted in the absence of any commercial or financial relationships that could be construed as a potential conflict of interest.

## Publisher's Note

All claims expressed in this article are solely those of the authors and do not necessarily represent those of their affiliated organizations, or those of the publisher, the editors and the reviewers. Any product that may be evaluated in this article, or claim that may be made by its manufacturer, is not guaranteed or endorsed by the publisher.
