# Supplementary material for: Functional and Structural Brain Connectivity in Children With Bilateral Cerebral Palsy Compared to Age-Related Controls and in Response to Intensive Rapid-Reciprocal Leg Training
Source: Front Rehabil Sci. 2022 Apr 5;3:811509. doi: 10.3389/fresc.2022.811509 (PMC9397804; doi:10.3389/fresc.2022.811509)
Supplement: Supplementary file 1 [file Table_1.DOCX]

**Supplemental Table 1: Correlations between PRE-intervention functioning and PRE-intervention regional fractional anisotropy.**

| **Correlate** | **Posterior thalamic radiation mean FA** | **Sagittal striatum mean FA** | **Middle cerebellar peduncle mean FA** | **Corticospinal tract FA** |
| --- | --- | --- | --- | --- |
| **Age** | **0.57** | **0.63*** | 0.52 | **0.74*** |
| **GMFCS level (I,II,III)** | -0.07 | -0.08 | -0.2 | -0.02 |
| **Elliptical (RPM free)** | 0.26 | 0.25 | 0.36 | 0.33 |
| **Cycle (RPM free)** | 0.31 | -0.01 | 0.04 | -0.18 |
| **Elliptical (RPM fast)** | 0.48 | 0.48 | **0.55** | 0.44 |
| **Cycle (RPM fast)** | 0.34 | 0 | -0.04 | -0.09 |
| **Gait velocity (free; m/s)** | 0.25 | 0.34 | 0.22 | 0.18 |
| **Gait velocity (fast)** | 0.06 | 0.07 | 0 | 0.15 |
| **Gait cadence (free)** | 0.42 | 0.5 | 0.42 | 0.31 |
| **Gait cadence (fast)** | -0.03 | -0.25 | -0.23 | -0.21 |
| **KE Torque 30** | 0.26 | 0.23 | 0.02 | 0.18 |
| **KE Torque 90** | -0.04 | -0.11 | -0.27 | -0.11 |
| **SCALE score** | **0.59** | 0.26 | 0.21 | 0.37 |
| **PODCI Global Score** | 0.22 | 0.22 | 0.31 | 0.15 |
| **PODCI Transfers** | 0.22 | 0.08 | 0.19 | -0.02 |
| **PODCI Sports** | -0.05 | -0.09 | -0.04 | -0.21 |
| **PEDI Self-care** | 0.44 | 0.37 | 0.47 | 0.38 |
| **PEDI Mobility** | 0.09 | 0.08 | 0.32 | -0.01 |
| **Trained-modality change score (RPM free)** | -0.02 | 0.22 | 0.3 | 0.21 |
| **Trained-modality change score (RPM fast)** | -0.2 | -0.09 | -0.32 | -0.32 |

Table entries indicate Spearman correlation coefficients between regional white matter mean fractional anisotropy values (columns) and measures of age/function (rows). All rows except for the bottom two (shaded) rows indicate PRE-intervention measures; the shaded rows instead indicate PRE- to POST-intervention change in trained-device cadence. Bolded/highlighted correlations indicate those with absolute values greater than $\sqrt{0.3}$ (0.54); i.e., those estimated to share at least 30% variance in either a positive (gold background) or negative correlation (pink background). Significant correlations are additionally indicated with an asterisk. Mean FA was positively associated with age in the sagittal striatum and in the corticospinal tract. There was a trend toward a positive association between posterior thalamic radiation FA and SCALE scores.

**Table 3: Correlations between PRE-intervention functioning and PRE-intervention resting-state functional connectivity.**

| **Correlate** | **Correlation (⍴) vs. PRE-intervention PreC-Thalamus RSFC** | **Correlation (⍴) vs. PRE-intervention PreC-Midbrain RSFC** |  | **Correlation (⍴) vs. PRE-intervention PreC-Pons RSFC** |
| --- | --- | --- | --- | --- |
| **Age** | -0.07 | 0.49 |  | -0.18 |
| **GMFCS level (I,II,III)** | 0.14 | -0.54 |  | -0.25 |
| **Elliptical (RPM free)** | 0.04 | 0.51 |  | 0.04 |
| **Cycle (RPM free)** | -0.20 | 0.51 |  | 0.26 |
| **Elliptical (RPM fast)** | -0.10 | **0.61** |  | 0.03 |
| **Cycle (RPM fast)** | -0.30 | 0.48 |  | 0.08 |
| **Gait velocity (free; m/s)** | -0.04 | **0.62** |  | 0.22 |
| **Gait velocity (fast)** | -0.31 | 0.23 |  | 0.00 |
| **Gait cadence (free)** | 0.02 | 0.48 |  | 0.29 |
| **Gait cadence (fast)** | -0.07 | -0.09 |  | 0.52 |
| **KE Torque 30** | -0.31 | 0.16 |  | -0.09 |
| **KE Torque 90** | -0.32 | -0.02 |  | -0.20 |
| **SCALE score** | -0.27 | 0.10 |  | -0.18 |
| **PODCI Global Score** | 0.14 | **0.63** |  | 0.13 |
| **PODCI Transfers** | -0.01 | **0.63** |  | 0.27 |
| **PODCI Sports** | -0.02 | **0.60** |  | 0.21 |
| **PEDI Self-care** | -0.02 | **0.61** |  | 0.08 |
| **PEDI Mobility** | 0.09 | **0.69*** |  | 0.28 |
| **Trained-modality change score (RPM free)** | -0.51 | -0.45 |  | -0.37 |
| **Trained-modality change score (RPM fast)** | -0.36 | 0.07 |  | -0.46 |

Table entries indicate Spearman correlation coefficients between inter-regional functional connectivity values (columns) and measures of age/function (rows). All rows except for the bottom two (shaded) rows indicate PRE-intervention measures; the shaded rows instead indicate PRE- to POST-intervention change in trained-device cadence. Bolded/highlighted correlations indicate those with absolute values greater than $\sqrt{0.3}$ (0.54); i.e., those estimated to share at least 30% variance in either a positive (gold background) or negative correlation (pink background). Significant correlations are additionally indicated with an asterisk. Higher functional connectivity between precentral cortex and midbrain was associated with greater PEDI Mobility scores.
