# Supplementary material for: Functional and Structural Brain Connectivity in Children With Bilateral Cerebral Palsy Compared to Age-Related Controls and in Response to Intensive Rapid-Reciprocal Leg Training
Source: Front Rehabil Sci. 2022 Apr 5;3:811509. doi: 10.3389/fresc.2022.811509 (PMC9397804; doi:10.3389/fresc.2022.811509)

**Supplemental Figure 1: Volumetric analysis (resolution levels 1,2,4,5).**


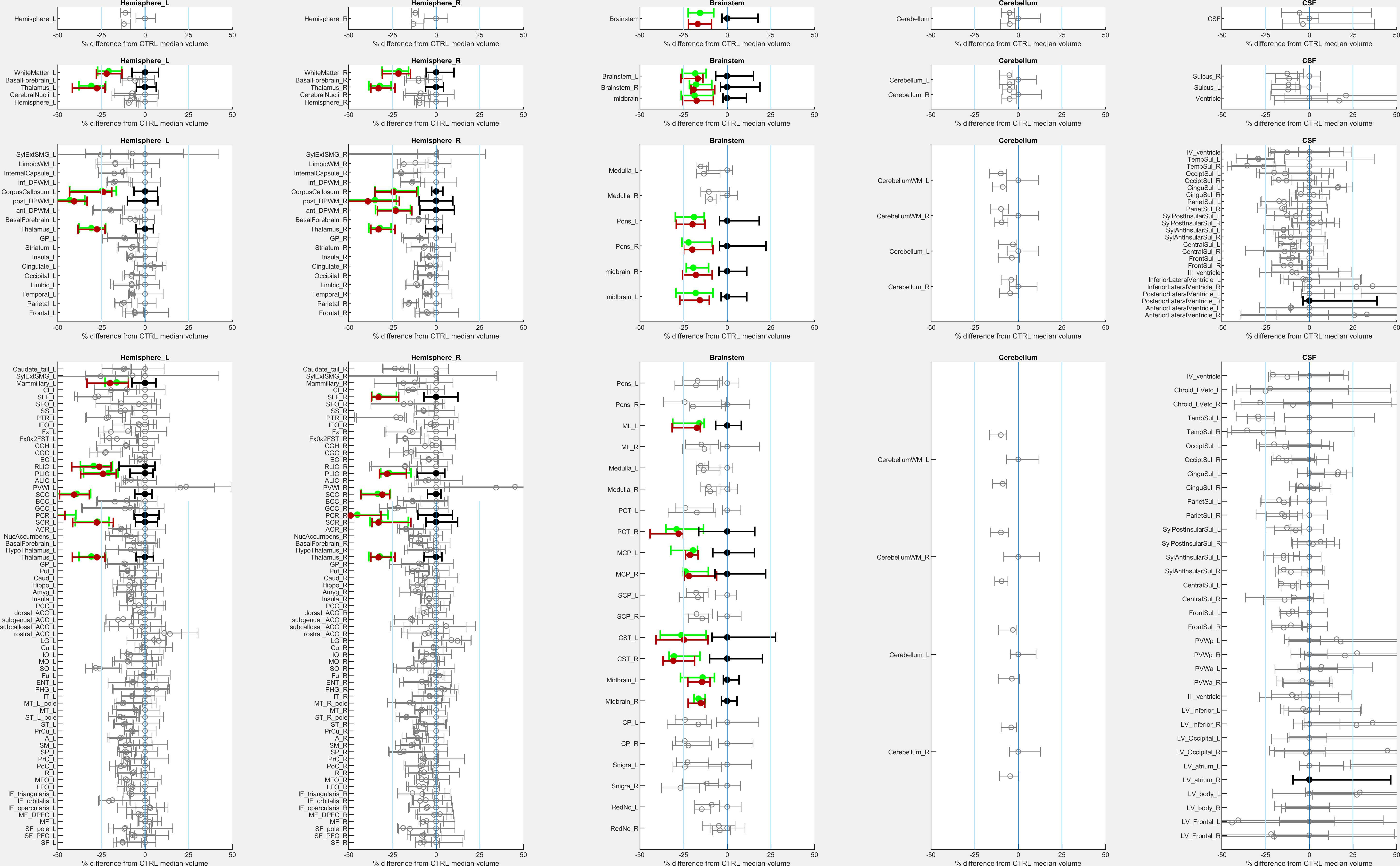


Panel rows indicate level of resolution (rows from top to bottom: Level 1=largest ROIs, Level 2, Level 4, then Level 5=finest ROIs). Panel columns indicate gross anatomical location as in **Figure 1** (columns from left to right indicating left hemisphere, right hemisphere, brainstem, cerebellum, and CSF structures, respectively). For each region of interest (ROI), error bars indicate median and 95% confidence intervals of ROI volumes for children with CP PRE-intervention (top in each triplet of errorbars), children with CP POST-intervention (bottom in each triplet), and CTRL children (middle of each triplet). X-axis volumes are normalized in terms of % difference from median CTRL ROI volume. Bolded/colored error bars (green = CP PRE-intervention, red = CP POST-intervention, and black = CTRL) indicate significant group differences surviving Bonferroni-Holm correction (applied within each Level). ROI designations reflect those of the MRIcloud MPRAGE atlas**.**

**Supplemental Figure 2: CTRL vs. CP resting-state BOLD functional connectivity: All connections.** Functional connectivity was assessed in a non-selective manner including both *intra*-hemispheric connections (right panel) as well as *inter*-hemispheric connections (left panel). No differences survived multiple comparisons correction; colors indicate estimated standard effect size (Glass’s Δ). The white-outlined rectangle highlights cortical-deep gray sensorimotor connections **(inset)**; estimated Δ values are shown in inset cells. Groupwise functional connectivity in sensorimotor connections did not grossly change PRE- vs. POST-intervention.


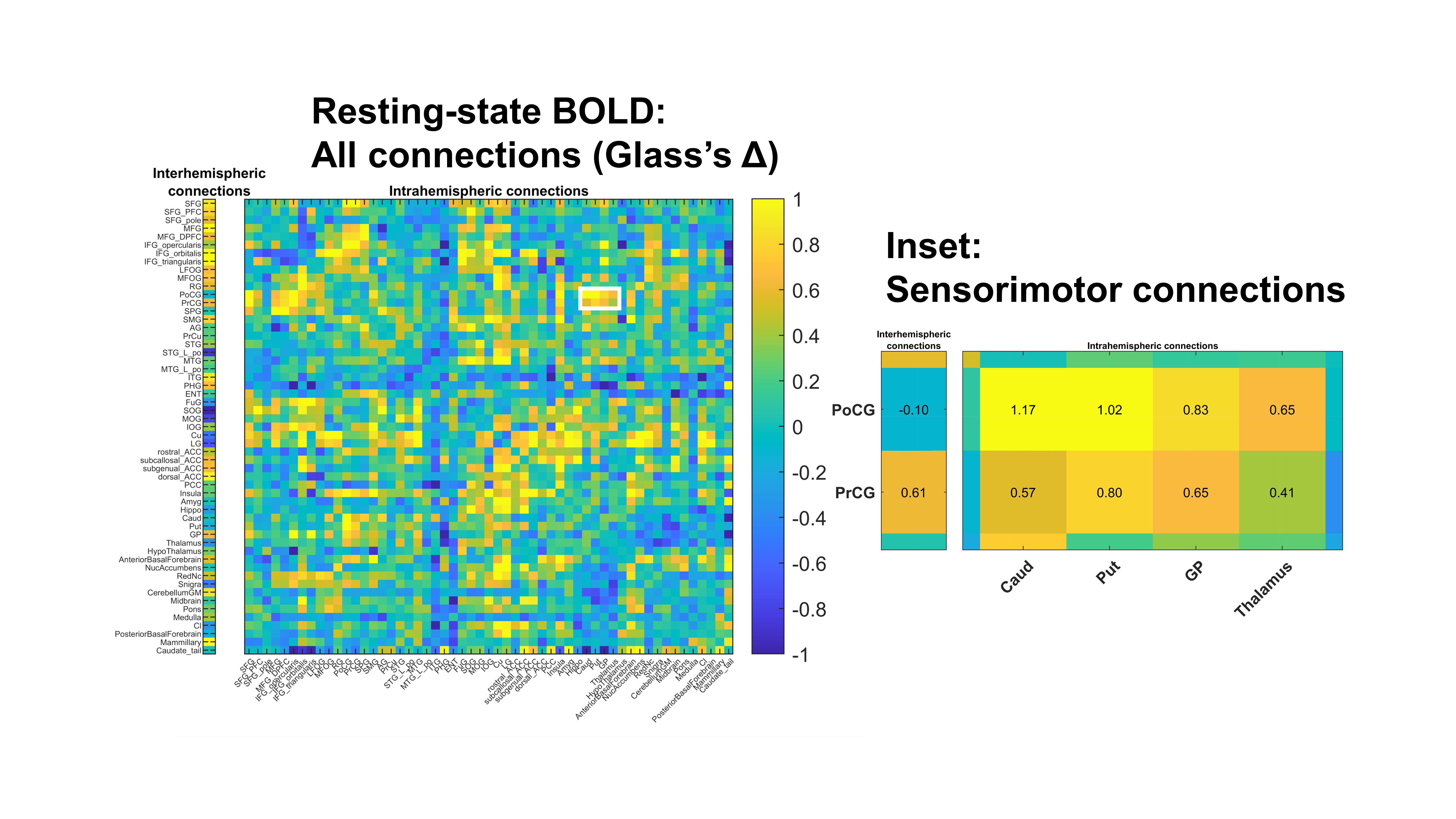

Supplement: Supplementary file 2 [file Data_Sheet_1.docx]
